# Evaluating the Impact of a Teaching Program on the Knowledge, Quality of Life, and Symptoms Among Asthmatic Children

**DOI:** 10.7759/cureus.81022

**Published:** 2025-03-23

**Authors:** Deepthi Chakravarthy, Shuba Sankaranarayanan, Nirmala V, Anita David

**Affiliations:** 1 Child Health Nursing, MES College of Nursing, Perinthalmanna, IND; 2 Pediatric Medicine, Sri Ramachandra Institute of Higher Education and Research, Chennai, IND; 3 Psychiatric Nursing, MES College of Nursing, MES Medical College Hospital, Perinthalmanna, IND; 4 Paediatric Nursing, Sri Ramachandra Institute of Higher Education and Research, Chennai, IND

**Keywords:** asthma, asthma symptom, asthmatic children, knowledge, quality of life, teaching program

## Abstract

Asthma is a prevalent chronic respiratory condition that affects millions of children worldwide, often disrupting their daily activities, academic performance, and overall well-being. Despite its impact, many children, along with their families and caregivers, have limited knowledge about asthma, its triggers, and effective management strategies. Implementing a structured teaching program is crucial to improving asthma awareness, fostering self-management through action plans, minimizing emergency incidents, and empowering children with the confidence and independence needed to manage their condition effectively.

The specific objectives were to assess the baseline levels of knowledge, quality of life, and asthma symptoms, and to determine the effect of the teaching program in improving these outcomes.

This randomized controlled trial was conducted among 94 participants from an asthma clinic, with 47 subjects in the experimental group and 47 in the control group, using an allocation concealment technique. The study assessed participants' knowledge, quality of life, and asthma symptoms using the Asthma Knowledge Questionnaire, the Mini Pediatric Asthma Quality of Life Questionnaire (MiniPAQLQ), and the Asthma Control Questionnaire (ACQ). After administering a pretest to both groups, the experimental group received structured education on various aspects of asthma management. Post-intervention assessments were conducted four times at four-week intervals using the same questionnaires as the pretest.

The collected data were tabulated and analyzed using descriptive and inferential statistics. The findings of the study indicate that the teaching program effectively improved participants' knowledge, quality of life, and asthma symptoms. The effectiveness of the intervention was assessed using repeated measures ANOVA. Posttest results demonstrated a statistically significant improvement in knowledge, quality of life, and asthma symptoms (P < 0.001).

The study concludes that an educational program on various aspects of asthma can help children with asthma better understand their condition, reduce anxiety, enhance coping mechanisms, and encourage responsibility in managing their disease. Therefore, alongside routine medical treatment, asthma education plays a crucial role in improving children's knowledge, quality of life, and symptom management.

## Introduction

Patients, their families, and communities are all greatly impacted by asthma, a common and potentially dangerous chronic illness. It is thought to impact over 300 million individuals globally [1]. The symptoms of asthma, a common respiratory disease in children, include coughing, chest tightness, wheezing, and shortness of breath. By enhancing their inhaler technique and their ability to identify and react to symptoms, children with asthma may be able to better manage their condition. Schools provide an environment that may be helpful for implementing interventions meant to help kids become better self-managers. Children with asthma who do not frequently visit primary care can be reached more easily thanks to the educational ethos, which also supports the acquisition of skills and knowledge. Given the complexity of self-management interventions, it is important to comprehend the combination of intervention characteristics linked to the effective implementation of asthma self-management programs [2].

It was discovered that asthma was more common in cities due to increased air pollution and among boys. At the primary healthcare level, it is essential to offer the right training and resources to guarantee early detection and efficient management. Furthermore, to get precise estimates of the asthma burden, a national population-based survey is advised [3].

To better understand asthma, including medication options, triggers to avoid, and at-home symptom management, people with the condition and their families require information. To avoid a severe attack, people with asthma must understand how to modify their therapy when their symptoms worsen. An asthma action plan may be provided by healthcare professionals to provide patients greater control over their care. The United Nations 2030 Agenda for Sustainable Development and the World Health Organization's Global Action Plan for the Prevention and Control of Non-Communicable Diseases (NCDs) both acknowledge asthma [4].

Asthma frequently starts in childhood, however it can appear at any age. Childhood asthma symptoms are a reliable indicator of long-term consequences. Many cases that last throughout adolescence carry over into adulthood, especially when they are linked to atopy, decreased lung function, and increased airway hyperresponsiveness [5]. Even with the growing number of efficient treatments, asthma control is still not at its best. Asthma control and patient quality of life are also greatly impacted by non-pharmacological factors such as stress, allergens, and air pollution. Nevertheless, there hasn't been a thorough analysis of how well non-pharmacological treatments work to improve asthma management. Finding non-pharmacological treatments that are most likely to improve asthma management and overall control is the goal of this evaluation [6].

Children and teenagers in school are susceptible to bronchial asthma, a common and potentially fatal illness. Asthma flare-ups can cause absenteeism from school and impairment in everyday functioning. Inadequate understanding, incorrect inhaler technique, non-compliance with therapy, and bad attitudes regarding the condition and drugs are some of the factors that may make these issues worse [7]. Healthcare providers must follow evidence-based, stepwise drug administration based on symptom intensity, provide thorough patient education and develop customized asthma action plans for optimal asthma management. Additionally, effective interprofessional communication is crucial for ensuring seamless care coordination across healthcare settings and keeping all team members informed about acute exacerbations, ED visits, hospital admissions, and medication changes [8]. In order to prevent exacerbations and provide the best possible control of the condition, patient education is essential in the therapy of pediatric asthma. Teaching children and caregivers about asthma causes, symptoms, and the need to follow treatment regimens are essential elements of this approach. Patients and caregivers need to be able to recognize the early warning indicators of exacerbations and know when and how to get help right away. Teaching the correct use of inhalers, spacer devices, and monitoring methods such as peak flow measurements enhances confidence in managing asthma at home. Talking to caregivers about possible asthma drug side effects is also essential since it gives them the knowledge they need to identify negative reactions and make decisions about their child's care [9].

Information alone is not as effective as structured education initiatives. With the use of resources such as individualized written action plans, symptom assessments, and/or peak expiratory flow measurements, these programs concentrate on educating patients on how to effectively manage their asthma on their own. They considerably enhance the management of asthma when paired with routine medical checkups. All asthma sufferers and their families should be introduced to these programs at an early age. They can be given as part of a larger care network, during consultations, or while a patient is in the hospital [10].

One of the main causes of the high morbidity rates among asthma patients is a lack of understanding regarding asthma management and first-aid procedures. It is anticipated that health education will improve people with asthma's understanding of and proficiency in first aid [11]. Patients who get asthma education can enhance lung function and asthma management, lessen the likelihood of nighttime respiratory problems and asthma attacks, and cut down on hospital stays, ER visits, unplanned doctor's appointments, and lost work or school days. In the end, it helps people with asthma live better lives [12].

Many children and their parents lack sufficient knowledge about asthma triggers, medication administration, and emergency response. Children who are educated about asthma are more equipped to understand and take charge of their condition. It boosts their confidence in symptom management and trigger recognition. Effective training can reduce the frequency and severity of asthma attacks.

## Materials and methods

A quantitative randomized controlled trial design was used in this investigation. The study was conducted at the asthma clinic of MES Medical College Hospital, located in the Malappuram district of Kerala, India. A total of 94 known cases of asthmatic children participated in the study, divided into an experimental group (n=47) and a control group (n=47). Using simple random sampling (coverslip approach), eligible children who satisfied the inclusion requirements were randomized to either the experimental or control group. 

This study was conducted in Kerala, India where the predominant language is Malayalam. However, all participating children were proficient in reading English, which facilitated the data collection process. The research tool was constructed in both Malayalam and English languages, the participants have to be able to read both Malayalam and English and be between the ages of 10 and 17. The study did not include children with developmental or cognitive problems or those who had severe asthma flare-ups.

Sample size

The sample size was calculated for doing repeated measures of ANOVA, which came out to be 94 participants overall (47 in each group). The objective was based on detecting a clinically relevant difference of 0.5 in asthma control as statistically significant between the two groups. The Z-value corresponding to the α error (Z_{α/2}) was 1.96, while the Z-value corresponding to the β error (Z_{β}) was 0.84. The average standard deviation (SD) of asthma control was calculated as (SD₁ + SD₂)/2, where SD₁ and SD₂ were the standard deviations in each group (0.85, averaged from 0.95 and 0.75). Using the formula below, the required sample size was determined to be 47 participants per group. Therefore, the total sample size required for the study was 94 participants, with 47 allocated to the intervention group and 47 to the control group.

$N = (Z_{&alpha;/2} + Z_{&beta;})^2 * (SD^2 / 2d^2)$

Procedure

The investigation was carried out at the pediatric asthma clinic of a particular hospital. The experimental group was given a designed educational session, the teaching was administered individually to each participant for a single 20-minute session including a variety of asthma-related topics. The control group continued receiving their usual medical care and received no intervention. This study employed three questionnaires: the Asthma Control Questionnaire (ACQ), the Mini Pediatric Asthma Quality of Life Questionnaire (Mini PAQLQ), and an Asthma Knowledge Questionnaire. 

The questionnaires were prepared and submitted to subject experts for validation and review. The three parts of the Asthma Knowledge Questionnaire were as follows: the first part gathered the child's demographic information, the second part evaluated the child's understanding of asthma, and the third part examined respiratory outcomes. The 13 questions of the standardized Mini Pediatric Asthma Quality of Life Questionnaire are divided into three domains: emotional functions (four questions), symptoms (six questions), and activity limits (three questions). It uses a 7-point Likert scale, with 7 denoting a high quality of life in terms of health and 1 denoting a low quality of life. There are seven items on the Asthma Control Questionnaire (ACQ). Peak expiratory flow (PEF) percentage is measured in the seventh item, while six items use a six-point scale to evaluate the child's asthma experiences over the previous seven days (0 being total control and 6 being no control) [13]. To monitor progress, the pre-test data collection tool was reused at three post-test time points, spaced four weeks apart, for both the experimental and control groups.

Intervention

For children with asthma, a planned health education is prepared. The researcher used flashcards containing definitions, causes, risk factors, pathophysiology, clinical features, medical management, and prevention regarding asthma as a teaching tool to instruct the experimental group members. Each child received a single 20-minute session, which was supplemented with a take-home pamphlet for further reference. This health teaching will help to raise knowledge, quality of life, and symptom control.

Data collection

The data was gathered by the researcher from February 2023 to March 2024 at MES Medical College Hospital, Malappuram District. Participants completed researcher-assisted, self-administered questionnaires to assess their quality of life, symptom control, and asthma knowledge. After completing the questionnaire, the child's respiratory outcomes like PEF and respiratory rate were evaluated and recorded. The researcher then instructed the experimental group using flashcards. The same data collection tool used for the pre-test was administered three times during the post-test period, at four-week intervals, to track progress in both the experimental and control groups.

Data analysis

The demographic information in this study was compiled using descriptive statistics. Multiple observations of asthma knowledge, quality of life, and symptom control over time were compared using repeated measures analysis of variance (ANOVA).

Ethical consideration

The study was approved by the institutional ethics committee and registered with the Clinical Trials Registry of India (Reg. No. CTRI/2021/05/043488). The researcher obtained informed consent from the parents or guardians and assent from the participants who were willing to take part in the study.

## Results

There were 23 (49%) females and 24 (51%) males in the control group, compared to 21 (45%) females and 26 (55%) males in the experimental group. Among children with asthma, 31 (66%) were in the experimental group and 32 (68%) were in the control group. In the experimental group, 30 (64%) participants were from metropolitan areas, and 17 (36%) were from rural areas. In contrast, the control group included 27 (57%) participants from urban areas and 20 (43%) from rural areas. The majority of participants belonged to the Muslim religion. A family history of asthma was reported in 15 (32%) of the control group and 14 (30%) of the experimental group. Most individuals in both groups had been suffering from asthma for six to 10 years. To manage their symptoms, 46 (98%) of the experimental group and 45 (96%) of the control group used asthma medication. 

**Table 1:** Frequency distribution of background between the experimental and control group

Demographic Characteristics		Experimental group (n=47)		Control group (n=47)		Total (n=94)	
		Frequency	Percentage	Frequency	Percentage	Frequency	Percentage
Age in years	10-13	32	68	35	74	94	100
	14-17	15	32	12	26		
Sex	Male	26	55	24	51	94	100
	Female	21	45	23	49		
Education	Upper primary	31	66	32	68	94	100
	High school	14	30	13	28		
	Higher secondary school	02	04	02	04		
Residential Area	Urban	30	64	27	57	94	100
	Rural	17	36	20	43		
Religion	Hindu	12	25	15	32	94	100
	Muslim	29	62	27	57		
	Christian	06	13	05	11		
Family history of asthma	Yes	14	30	15	32	94	100
	No	33	70	32	68		
Time since diagnosis (in years)	<1	0	0	02	04	94	100
	1-5	11	24	12	26		
	6-10	34	72	32	68		
	11-15	02	4	01	02		
Usage of asthma medicine	No	01	02	02	04	94	100
	Yes	46	98	45	96		

Table 2 demonstrates that the mean knowledge levels of the experimental and control groups varied significantly at different times, as shown in the above table. In particular, there was a significant group difference (F-value = 153.01) in the experimental group's mean knowledge level from the pre-test (mean value: 10.55) to the final visit (mean value: 24.06) when comparing the effectiveness of the two groups. A repeated measures ANOVA test was used to confirm the substantial difference between the control and experimental groups at different time periods, and the results showed that the difference was significant at the (P < 0.001) level. This suggests that the educational program was successful in increasing the children with asthma's understanding of the condition.

**Table 2:** Analysis of effect of teaching program on level of knowledge

Group	Knowledge level, mean(SD)					
	Pre-test	Post-test 1	Post-test 2	Post-test 3	F-value	P-value
Control group (n=47)	11.38(3.97)	12.72(3.99)	13.11(3.84)	14.15(4.42)	153.01	<0.001
Experimental group (n=47)	10.55(4.42)	21.15(4.37)	23.6(4.08)	24.06(4.09)		
Total	10.97(4.2)	16.94(5.94)	18.35(6.58)	19.11(6.54)		

The impact of an asthma education program on the quality of life of children with asthma was evaluated using repeated measures ANOVA, as shown in Table 3. The findings showed that the experimental and control groups' quality of life differed statistically significantly (F = 30.63, p < 0.001). These results suggest that the asthma education program significantly improved the quality of life for kids with asthma. 

**Table 3:** Effect of teaching program on quality of life.

Groups	Pediatric asthma quality of life, mean (SD)					
	Pre-test	Post-test 1	Post-test 2	Post-test 3	F-value	P-value
Control group (n=47)	3.24(0.39)	3.3(0.4)	3.38(0.36)	3.4(0.39)	30.63	<0.001
Experimental group (n=47)	3.32(0.5)	3.4(0.54)	3.46(0.49)	3.5(0.5)		
Total	3.28(0.45)	3.34(0.5)	3.42(0.43)	3.45(0.45)		

When the mean values of the two groups are compared at different time intervals, Table 4 demonstrates that the experimental group's mean value (mean=3.1) is lower than the control group's (mean=3.4). This implies that symptoms are under the experimental group's control (F = 17.99, p < 0.001). These findings suggest that the instructional program was beneficial in improving asthma management.

**Table 4:** Effect of teaching program on asthma control

Groups	Asthma control, mean (SD)					
	Pre-test	Post-test 1	Post-test 2	Post-test 3	F-value	P-value
Control group (n=47)	3.39(0.49)	3.52(0.37)	3.35(0.39)	3.39(0.39)	17.99	<0.001
Experimental group (n=47)	3.42(0.66)	3.24(0.46)	3.14(0.62)	3.08(0.66)		
Total	3.50(0.59)	3.38(0.44)	3.25(0.52)	3.23(0.57)		

## Discussion

The current study found that the majority of participants were in the 10-13 age range, belonged to the male gender, and lived in urban areas. Both groups had a similar distribution in education level and religion, with most identifying as Muslim. The participants in both groups had a diagnosis of asthma for 6-10 years, with minimal family history of the condition. The majority of participants were currently using asthma medications.

The study shows that at different times, the mean knowledge levels of the experimental and control groups varied considerably. The experimental group's mean knowledge level significantly rose from the first visit (mean: 10.55) to the final visit (mean: 24.06), indicating that the teaching program was effective. The two groups showed a substantial difference (F = 153.01, P < 0.001). These results show that the educational program was successful in raising asthmatic children's awareness of the condition. A systematic review was carried out to investigate the effects of asthma education programs on children's self-management, quality of life, school absenteeism, and asthma knowledge in order to support these findings. A systematic review and meta-analysis evaluated how asthma education affected four important domains: self-management, school absenteeism, quality of life, and children's understanding of asthma. The findings suggest that asthma education initiatives improve outcomes in these domains for children with asthma [14].

The impact of an education program on the quality of life of children with asthma was assessed in this study using repeated measures ANOVA. The findings showed that the experimental and control groups' quality of life differed significantly (F = 30.63, p < 0.001). These results imply that the asthma education program was successful in raising the standard of living for kids with asthma. A comparable study was carried out to assess the efficacy of a mobile health education application in improving the quality of life among pupils with asthma in urban Malaysia during the COVID-19 pandemic. In this quasi-experimental study, which employed a pre-and post-intervention design, 214 students were randomized to either the intervention or the control group. The control group received health education in person, while the intervention group received it through a mobile application. The findings demonstrated an improvement in the quality of life for both groups. The control group's mean overall score increased from 5.31 ± 1.27 prior to the intervention to 5.66 ± 1.28 following it. The experimental group's mean overall score rose from 5.01 ± 1.36 to 5.85 ± 1.29. An independent t-test comparison revealed a statistically significant difference in the mean quality of life scores between the two groups [15].

According to the findings, most individuals in the experimental group had better asthma control than those in the control group (F = 17.99, p < 0.001). These results imply that the instructional program improved participants' asthma control. In this study, the researcher delivered a teaching program utilizing flashcards with pictures to facilitate an easy understanding of various asthma-related concepts among children. A study from Turkey was conducted to assess the impact of the Health Promotion Program for Children with Asthma (HPPCA) on the quality of life and illness control in children. Drawing on brain-based learning theories and Nola J. Pender's Health Promotion Model, the curriculum used cartoons and colouring materials. The study sample consisted of 74 children aged 7 to 11 who were treated at Istanbul University Hospital's respiratory illnesses unit. At both the first- and fourth-month follow-ups, the experimental group's children had significantly higher asthma control and quality of life scores than the control group (p <.001). These results imply that the HPPCA education program is successful in raising children with asthma's quality of life and controlling their asthma [16].

This study highlights the benefits of targeted education for asthmatic children, leading to improved disease management, reduced symptoms, and enhanced quality of life. Access to age-appropriate education and counselling is essential for supporting these children and promoting optimal health outcomes.

## Conclusions

Since asthma is a chronic condition that cannot be cured, it must be managed for a better quality of life. Raising awareness of asthma in children is crucial to helping them take greater responsibility for their illness. According to the study's findings, teaching kids how to manage their asthma greatly enhances their understanding, quality of life, and ability to control their symptoms. Optimal asthma control and improved health outcomes necessitate sustained educational efforts. Effective asthma management in children can be achieved through integrating educational programs in schools and promoting parental understanding, thereby fostering a supportive environment for better disease management.
